# Navigating Personal, Social, and Environmental Obstacles to Healthy Lifestyle in Jordanian Adolescents

**DOI:** 10.1155/tswj/8889672

**Published:** 2025-01-30

**Authors:** Rula A. Amr, Ahmed M. Al-Smadi, Nanda Alqutob, Rand T. Akasheh

**Affiliations:** ^1^Department of Nutrition and Health Psychology, American University of Madaba, Madaba, Jordan; ^2^Nursing Department, Fakeeh College for Medical Sciences, Jeddah, Saudi Arabia; ^3^Department of Internal Medicine, The Ohio State University, Columbus, Ohio, USA; ^4^Department of Pediatrics, University of Illinois at Chicago, Chicago, Illinois, USA

**Keywords:** adolescents, healthy eating, perceived barriers, physical activity

## Abstract

**Objective:** This study aimed to investigate barriers hindering healthy eating and physical activity among Jordanian adolescents.

**Methods:** A random sample of 1040 adolescents (596 males and 444 females) aged 11–17 from various Jordanian schools participated. A nutritionist-administered questionnaire assessed barriers.

**Results:** The study unveiled diverse personal, social, and environmental barriers to healthy lifestyles. Notably, inadequate knowledge of nutrition and limited time for healthy food preparation due to homework emerged as key barriers to healthy eating. Conversely, insufficient exercise skills and reduced enjoyment of physical activity were the primary impediments to physical activity. Females perceived homework as a more substantial barrier than males (*p*=0.027).

**Conclusion:** These barriers may contribute to increasing childhood obesity rates in Jordan. Future interventions should prioritize creating a supportive environment that respects cultural norms, emphasizing high-quality parks, nutrition and sports education, healthier food options, student motivation, and park visits. Community engagement is crucial for fostering healthier lifestyles among Jordanian youth.

**Summary**

• This study sheds light on the significant barriers to healthy eating and physical activity among Jordanian adolescents, highlighting the urgent need for tailored interventions and policies.
• By emphasizing the importance of high-quality parks, nutrition and sports education, and healthier food choices, this research contributes to the fight against rising childhood obesity rates in Jordan.

## 1. Introduction

Lifestyle-related diseases or noncommunicable diseases (NCDs), such as cardiovascular diseases (CVDs), cancers, stroke, and diabetes, are among the leading causes of mortality worldwide [1]. In 2016, NCDs accounted for 72% of total global deaths that year [2]. Overweight and obesity are major risk factors for NCDs, in addition to poor dietary habits, lack of physical activity, tobacco use, dyslipidemia, and high blood pressure [3]. In 2014, the Eastern Mediterranean Region (EMR) had estimated overweight and obesity rates above the global average, with a high proportion of adolescents in the Arab population experiencing overweight or obesity [1]. As a result, prevalence rates are expected to reach epidemic levels, leading to high rates of morbidity and mortality [3].

In Jordan, youths constitute the majority of the population, with 68.8% of them being under 25 years of age. Adolescents (11–17 years) make up 24% of the Jordanian youth population (under 25 years old) [4]. According to the Global School-Based Student Health Survey (GSHS) implemented in Jordan in 2007, 14.3% of boys and 3.9% of girls in adolescence experienced overweight or obesity, respectively [5]. A later study conducted in 2016 reported that 46% of girls in adolescence residing in Amman—the capital of Jordan—had obesity and overweight and found that more than 50% of Jordanian adolescents were experiencing malnutrition [6]. Adolescent overweight or obesity can negatively impact quality of life and lead to long-term health consequences, such as high blood pressure, dyslipidemia, type II diabetes, CVD, and psychological problems [4].

During adolescence, diet plays a critical role in development; however, adolescents are also exposed to social pressures and seek to gain more autonomy, which can alter their behavior during this life stage [7]. This puts adolescents at risk of developing unhealthy habits, such as smoking, poor dietary habits, sedentary lifestyle, and alcohol abuse, which increases the risk of disease [7]. Jordan has undergone a nutritional transition alongside economic growth, urbanization, and dramatic improvements in living conditions. This transition has negatively impacted the health and lifestyle of its population, particularly children and adolescents [4]. These lifestyle changes are attributed to increased dependency on transportation, skipping breakfast, low intake of fruits and vegetables, frequent consumption of fast foods, and lack of physical activity due to prolonged television watching, computer game playing, and Internet use [6, 8, 9].

Given the high rate of obesity both globally and at the national level, as well as poor lifestyle habits, it is essential to study the barriers to a healthy lifestyle. Studies conducted in western countries demonstrated that barriers to a healthy lifestyle can be attributed to personal, social, cultural, and environmental factors [10, 11]. Similarly, studies in Arab countries have demonstrated that lack of information on healthy eating, lack of motivation to consume a healthy diet, and limited time to prepare healthy food are the major barriers to healthy eating among adolescents [12]. However, research on the barriers to adopting a healthy lifestyle is limited in Jordan. Therefore, it is crucial to understand the barriers that prevent Jordanian adolescents from adopting a healthy lifestyle that promote physical activity and healthy eating. Thus, the objectives of this study are to identify the most and least prevalent barriers to healthy eating among school students; identify the most and least prevalent barriers to physical activity among school students; and examine the gender differences in barriers to healthy eating and physical activity.

## 2. Methodology

### 2.1. Ethical Considerations

The current study was conducted according to the guidelines from the Declaration of Helsinki, and all procedures involving human subjects were approved by the American University of Madaba (AUM) Research Ethics Committee.

### 2.2. Study Design and Settings

The present study utilized a cross-sectional survey design to gather data from various locations in Jordan, including the capital city of Amman and the city of Madaba. Adolescents from both public and private schools in Jordan were invited to participate. Seven nutrition and dietetics students from the AUM assisted in recruiting and administering the questionnaire to participants. Data were collected in March and April 2022.

Participants in grades 6 through 12 were randomly selected from seven different schools in Jordan including *the Baptist School, National Orthodox School, Al-Manhal International School, Al-Rashad Ideal School, Ibn Taymeyah Secondary Comprehensive School, Al-Ridwan School, and First University School.* Prior to data collection, the purpose of the study was explained to each potential participant. Informed consent forms were signed by the parents of participating adolescents to ensure their comprehension and voluntary participation of their children in the study.

A power analysis was conducted using G∗Power software (Version 3.1.9.7) and revealed that a minimum sample size of 844 participants (422 females and 422 males) is needed to achieve a power level of 0.95 at an *α* of 0.05, based on assumed proportion difference between males and females in the frequency of barriers.

The final sample size comprised 1040 individuals between the ages of 11–17, representing a diverse range of national and cultural backgrounds, consisting of both males and females. All participants who were enrolled in this study completed the questionnaire. Thus, no dropouts were recorded.

### 2.3. Study Questionnaire

The questionnaire consisted of three pages and 47 questions divided into three sections: (1) demographics; (2) questions regarding physical activity and health status; (3) a survey to evaluate perceived barriers to healthy eating and physical activity.

The questionnaire on perceived barriers to healthy eating and physical activity was extracted from a validated Arabic self-reported questionnaire, which was used to evaluate perceived barriers among adolescents in seven Arab nations [12]. Permission to use the questionnaire was granted from the principal investigator. The questionnaire has two sections; the first section focused on barriers to physical activity, including 3 items on personal barriers, 3 items on social support barriers, and 8 items on environmental barriers. The second section focused on barriers to healthy eating, including 6 items on personal barriers and 4 items on social and environmental barriers. For each item in the perceived barriers survey, the participant rated the barrier as “*not a barrier*,” “a *somewhat important barrier*,” and “a *very important barrier*.”

### 2.4. Evaluating Physical Activity Level

Using the guidelines of the 2005 International Physical Activity Questionnaire (IPAQ) (14), physical activity was classified into high, moderate, and low level of physical activity. The *high-level physical activity* is defined as performing vigorous physical activity on ≥ 3 days/week to achieve at least 1500 metabolic equivalent (MET)-minutes weekly, or physical activity on ≥ 7 days of walking, moderate exercise, and/or vigorous activity to achieve ≥ 3000 MET-minutes per week of exercise. The *moderate-level physical activity* group is defined by vigorous activity for ≥ 20 min on ≥ 3 days/week, or moderate activity and/or walking for ≥ 30 min/day and on ≥ 5 days/week, or achieving 600 MET-minutes weekly through walking, moderate activity, and/or vigorous activity on ≥ 5 days/week. The *low-level physical activity* group was defined as not belonging to either of the high and moderate activity categories [13].

### 2.5. Statistical Analysis

Statistical analysis was performed using SPSS software (Version 24 for Windows 10; SPSS, 2014, Chicago, IL, USA). Descriptive analysis was used to calculate the baseline characteristics of study participants. The frequencies of the three possible responses to each item in the perceived barriers questionnaire (“*not a barrier*,” “a *somewhat important barrier*,” and “a *very important barrier*”) were calculated to find the strongest and weakest barriers to healthy eating and physical activity.

Additionally, the responses “*not a barrier*,” “a *somewhat important barrier*,” and “a *very important barrier*” were coded into 1, 2, and 3, respectively, to calculate the mean and standard error of mean (SEM) for each response. The mean responses were calculated to each item in the questionnaire, each subscale, and the overall scales. A chi-square test was used to examine the whether frequencies of perceived barriers were different in female versus male participants. Independent sample *t*-test was used to examine the differences in mean responses to perceived barriers between males and females. A *p* value of < 0.05 implied statistical significance.

## 3. Results

### 3.1. Demographic and Clinical Details

The sample for the study consisted of 1040 participants with a mean age of 15.11 (SD = 1.59) and ranging from 11 to 17 years old. As seen in Table 1, the majority of participants were male (*N* = 596, 57.3%), of Jordanian nationality (*N* = 906, 87%), residing in the city of Amman (*N* = 890, 85.6%), without chronic illness (*N* = 985, 94.7%), not taking medication (*N* = 988, 95.0%), nonsmokers (*N* = 939, 90.3%), and displaying low levels of physical activity (*N* = 565, 54.3%)

### 3.2. Perceived Barriers to Healthy Eating

Table 2 illustrates the participants' mean response to each question, ordered from highest to lowest. The highest-rated personal and environmental barriers to healthy eating were the lack of information about healthy nutrition (item 1) and the lack of motivation to eat healthy food (item 2), while the lowest-rated barrier was the unaffordability of healthy food (item 6). Overall, personal and environmental barriers were perceived as “very important,” “somewhat important,” and “not important” by an average of 34%, 40.2%, and 25.8% of the study participants, respectively.

In terms of the social barriers to healthy eating, the highest-rated barrier was the lack of time to prepare healthy foods due to homework (item 7) followed by the lack of encouragement from parents to eat healthy foods (item 8), while the lowest-rated barrier was the lack of encouragement from teachers to eat healthy foods (item 10). Overall, social barriers were perceived as “very important,” “somewhat important,” and “not important” by an average of 34%, 40.2%, and 25.8% of the study participants, respectively. In summary, an average of 33.6%, 38.2%, and 28.2% of the participants considered the barriers to healthy eating as “very important,” “somewhat important,” and “not important,” respectively. Thus, these data indicate that most adolescents in our study perceived many barriers that prevent them from eating healthy.

### 3.3. Perceived Barriers to Physical Activity

Table 3 presents the mean scores for each question related to barriers to physical activity, ordered from highest to lowest. The highest-rated personal barrier to physical activity was the lack of motivation for physical activity or sports (item 1) followed by dislike of physical activity or sports (item 2), while the lowest-rated barrier was the lack of skill for proper exercise (item 3). Overall, personal barriers were perceived as “very important,” “somewhat important,” and “not important” by an average of 36.2%, 33.9%, and 29.9% of the study participants, respectively.

In terms of social barriers, the highest-rated barrier was the lack of motivation from parents to exercise (item 4), followed by the lack of motivation from friends to exercise (item 5), while the lowest-rated barrier was the lack of motivation from teachers to exercise (item 6). Overall, social barriers were perceived as “very important,” “somewhat important,” and “not important” by an average of 30.6%, 37%, and 32.4% of the study participants, respectively.

The highest-rated environmental barriers to physical activity were the lack of time to exercise due to homework (item 7) followed by the lack of information on how to increase physical activity (item 8), while the lowest-rated barriers were the inability to exercise outside the house due to customs and traditions (item 14) and feeling ashamed when playing sports in front of others (item 13). Overall, social barriers were perceived as “very important,” “somewhat important,” and “not important” by an average of 33.6%, 38.2%, and 28.2% of the study participants, respectively.

In summary, an average of 30.9%, 35.3%, and 33.8% of the participants considered the barriers to healthy eating as “very important,” “somewhat important,” and “not important,” respectively. Thus, these data indicate that most adolescents in our study perceived many barriers that prevent them from being physically active.

### 3.4. Differences in Barriers Based on Gender

The frequencies of perceived barriers to physical activity and healthy eating were calculated in females and males, and the chi-square test was used to test the differences between these frequencies based on sex. The analysis showed that not finding the time to exercise because of homework was more frequently perceived as a “very important” barrier in females (39.6%) compared to males (32.4%). However, no significant differences were observed between females and males in the frequencies of any of the other perceived barriers (Table 4). Overall, these findings show that females were more likely to perceive lack of time because of homework as a barrier to exercise.

### 3.5. Differences in Barriers Based on BMI

Table 5 examines the perceived barriers to healthy eating and physical activity among adolescents in Jordan, categorized by their BMI groups, including those with normal weight, overweight, and obesity. The sample consisted of 730 individuals classified as having normal weight, 170 individuals classified as experiencing overweight, and 140 individuals classified as experiencing obesity.

#### 3.5.1. Perceived Barriers to Healthy Eating

When it came to personal and environmental barriers to healthy eating, we found several noteworthy patterns. Notably, 18.1% of individuals in the normal weight category reported that they did not have enough information about healthy nutrition, while this percentage was slightly higher among individuals in the overweight category (11.2%) and individuals in the category of people with obesity (19.3%), with *p* values indicating no significant differences (*p*=0.180). Similarly, 20.4% of individuals with normal weight lacked motivation to eat healthy food compared to 15.3% in the overweight category and 18.6% in the category of people with obesity (*p*=0.405). Enjoyment of healthy food showed a similar trend, with no substantial differences between the BMI categories. However, financial constraints appeared to be a significant barrier for individuals in the overweight and people with obesity categories, as 42.4% of those in the overweight category and 44.3% of those in the category of people with obesity reported being unable to buy healthy food due to its cost, compared to 18.1% in the normal weight category (*p*=0.031).

#### 3.5.2. Social Barriers to Healthy Eating

Regarding social barriers to healthy eating, we observed that parents' encouragement to eat healthy foods did not significantly differ across BMI categories. However, there was a notable difference in the influence of friends, where 41.8% of overweight individuals reported that their friends did not encourage healthy eating, compared to 28.2% in the normal weight group (*p*=0.049).

#### 3.5.3. Personal Barriers to Physical Activity

In terms of personal barriers to physical activity, motivation levels showed no substantial differences across categories of people with different BMIs. However, the enjoyment of physical activity or sports varied significantly, with 35.3% of people with obesity reporting not enjoying physical activity compared to 28.8% in the normal weight group (*p*=0.003).

#### 3.5.4. Social Barriers to Physical Activity

Social barriers to physical activity, such as the motivation provided by parents, friends, and teachers, did not exhibit significant differences between the BMI categories.

#### 3.5.5. Environmental Barriers to Physical Activity

Among environmental barriers to physical activity, several noteworthy findings emerged. Notably, the availability of suitable places for exercise and the affordability of sports facilities differed significantly across BMI categories. For example, 44.3% of individuals with obesity reported a lack of suitable places for exercise compared to 30.7% in the group of people with normal weight (*p*=≤0.001), and 41.2% of individuals with obesity found no places with suitable prices for sports, compared to 23.2% in the group of people with normal weight (*p*=0.013).

## 4. Discussion

Sedentary lifestyle and physical inactivity are rapidly growing risk factors for chronic diseases in Jordan and other Arab countries [3]. The current study aimed to identify the barriers to healthy eating and physical activity among adolescents in Jordan. Results indicate that there is a variety of personal, social, and environmental barriers to healthy eating and physical activity among adolescents in Jordan. Additionally, a significant difference was found between females and males in only one of the perceived barriers.

Previous studies in the Arab world have reported that dietary habits among adolescents are unhealthy, highlighting the importance of promoting healthy lifestyles that include healthy eating [14]. However, the current study found that lack of information related to healthy nutrition was a dominant obstacle to healthy eating. This barrier is also commonly reported among adolescents in western countries [15]. This may indicate that current nutrition education programs are inadequate, particularly in schools and mass media. Studies on the effectiveness of nutrition education programs in changing dietary habits in Jordan and the Arab world are limited, but some evidence suggests that the impact of these programs is low, possibly due to insufficient information provided, lack of specialized personnel, and failure to attract public attention [15]. Furthermore, it was noted that the school curricula in Arab countries do not address many local dietary habits that are linked to existing diet-related diseases [16]. Importantly, a previous study found that while adolescents had sufficient knowledge about healthy eating habits, they did not apply this knowledge in part due to easy access to nutritionally inappropriate foods [17]. Thus, this indicates that the food environment may outweigh the effect of nutrition knowledge on adolescent food choices and supports the need to improve the food environment in order to improve eating habits.

According to our research, a major obstacle for students was a lack of interest in healthy food. This aligns with the findings of another study that examined the primary factors contributing to unhealthy eating habits among adolescents, including a preference for nutritionally inadequate foods, peer influence, and easy access to unhealthy options. These findings suggest that dietary choices are not only solely based on personal preferences but also shaped by economic, social, and cultural factors [17].

The role of parents in shaping food choices and availability in the home is well established [18]. We found that 38.1% of adolescents perceived the lack of encouragement from their parents as a very important barrier to healthy eating. It is possible that lack of nutrition knowledge and busy work schedules in parents limit their ability to provide adequate supervision and guidance for their children's food habits [19]. In addition to parental influence, adolescents are particularly susceptible to peer influence on dietary habits. Other research has shown that healthy food intake is associated with the home environment and parents, while fast food is linked to friendships and socioeconomic status [20]. Thus, it is unsurprising that 30.6% of adolescents in our study perceived that lack of encouragement from friends as a very important barrier to healthy eating. Furthermore, lack of teacher encouragement was perceived as barrier to healthy eating by 28.1% of adolescents in our study, while the rest perceived this aspect as a less important or not important barrier. Studies in western countries demonstrated that teachers who exhibit unhealthy eating habits are often perceived as barriers to healthy eating in adolescents [21].

Interestingly, we found that the lack of time to exercise because of homework was more frequently perceived as a “very important” barrier in females compared to males. A previous study demonstrated that personal barriers to physical activity were more often perceived as very important by female versus male adolescents [12]. However, in our study, we found no significant differences in frequencies of perceived personal barriers between females and males. Nonetheless, all types of barriers that were prevalent were perceived as very important in at least 21% of the females and 23.7% of the males.

Importantly, 54.3% of the adolescents who participated in our study were identified as low active. Studies on factors associated with physical activity in the Arab world are limited [22]. The recent nutrition transition in many Arab countries has led to a more sedentary lifestyle, with increased use of transportation and increased time spent on computers, electronic devices, and video games. The current study in Jordan found that lack of exercise skills and not enjoying physical activity or sports were major perceived barriers among adolescents. Importantly, cultural and social norms in the Arab region often limit women's ability to engage in outdoor physical activity and exercise, as many families do not permit their girls to participate in outdoor activities for cultural and safety reasons. Furthermore, many families do not allow their girls to wear sportswear while exercising, but instead they have to wear traditional dress which is not comfortable for physical activity, and this plays a main role in discouraging them from exercising outdoors [23].

Barriers to healthy eating and physical activity among adolescents in Jordan may also be attributed to socioeconomic and cultural factors. Jordan's low economic status has led to a delayed recognition of the nutrition transition and slower changes in eating habits and lifestyle. This has contributed to relatively high rates of sedentary behaviors and obesity among adolescents, affecting their ability to adopt a healthy lifestyle. It is important to note that these factors may differ in Jordan compared to other countries, and the sociocultural differences should be taken into consideration [12].

In the assessment of the perceived barriers to healthy eating and physical activity among Jordanian adolescents, stratified by their BMI categories, several intriguing patterns emerged. It is noteworthy that across the three BMI categories, no significant differences were found in the reported lack of information about healthy nutrition, a lack of motivation to consume healthy foods, or a lack of enjoyment of healthy foods. These results align with some previous research, such as the study by Cardel et al., which found that perceived barriers to healthy eating did not significantly differ among adolescents with varying BMI statuses [24]. This consistency may suggest that adolescents, regardless of their weight category, face similar challenges when it comes to nutrition knowledge, motivation, and food preferences.

However, financial constraints appeared to be a pronounced barrier among adolescents with overweight and obesity, with a significantly higher proportion reporting difficulty affording healthy foods compared to their counterparts with normal weight. This finding concurs with a study by Desbouys et al., which highlighted the financial challenges faced by adolescents from lower socioeconomic backgrounds in accessing healthy foods [25]. The observed disparities underscore the need for targeted interventions that address economic inequalities in accessing nutritious food options among adolescents.

Surprisingly, parental encouragement for healthy eating showed no significant differences across BMI categories. This finding contrasts with the results of a study by Dialektakou and Vranas, which identified parental influence as a significant factor in adolescents' dietary choices [26]. The variation in findings may be attributed to cultural or contextual differences, emphasizing the importance of considering cultural factors when designing interventions to improve dietary habits among adolescents.

Conversely, we noted a significant difference in the influence of friends on healthy eating habits, with a higher percentage of overweight adolescents reporting a lack of encouragement from friends. This finding is consistent with the study conducted by Finnerty et al, which emphasized the role of peer influence in shaping adolescents' dietary behaviors [27]. The disparity in friends' influence across BMI categories highlights the need to target peer-based interventions, particularly for adolescents at risk of overweight or obesity.

Personal barriers to physical activity, including motivation and enjoyment, did not substantially differ across BMI categories, suggesting that these factors are not strongly associated with weight status among Jordanian adolescents. These results align with a study by Ng et al., which found no significant differences in motivation for physical activity across BMI groups [28]. This consistent pattern suggests that adolescents, irrespective of their weight category, may benefit from interventions aimed at enhancing intrinsic motivation and enjoyment of physical activity.

Social factors, such as motivation from parents, friends, and teachers, did not exhibit significant differences between BMI categories. This finding is consistent with a study by Bunke which found that social support for physical activity did not vary significantly among adolescents with varying BMI statuses [29]. These results suggest that social support networks may play a similar role in facilitating physical activity among adolescents, regardless of their weight category.

Among environmental barriers to physical activity, significant disparities were noted in the availability of suitable places for exercise and the affordability of sports facilities. Adolescents with obesity reported a higher prevalence of a lack of suitable places for exercise and fewer places with suitable prices for sports. These findings are in line with studies by Johnson and Robinson, which emphasized the limited access to affordable and suitable physical activity facilities for adolescents in certain communities [30, 31]. Addressing these environmental barriers is crucial for promoting physical activity and reducing obesity rates among Jordanian adolescents.

The study has strengths and limitations that are worth noting. A valid and reliable Arabic survey was utilized to gather data from adolescents; however, the majority of the data were collected from private schools rather than public schools, and some of the questions may have been more representative of private over public school students.

## 5. Conclusion

This study is the first to investigate and highlight some of the barriers that impede healthy eating and physical activity in Jordanian adolescents. An average of one-third of the adolescents in this study reported perceiving barriers to healthy eating and physical activity as very important factors that influence their lifestyles. Thus, the availability of unhealthy foods and lack of physical activity opportunities may be leading to high energy intake and low energy expenditure among Jordanian adolescents and might explain the rising rate of childhood obesity in Jordan. Future studies must focus on designing interventions and policies that eliminate or help overcome these barriers.

## 6. Recommendations

Based on our findings, we offer a comprehensive roadmap for policymakers to promote healthy lifestyles among children and adolescents. This roadmap comprises specific steps to bolster the availability of high-quality parks, integrate nutrition and sports education into school curricula, provide healthier food choices in school canteens, incentivize student participation in physical activity, and encourage parents to frequent parks with their children.

In Jordan, where the scarcity of community parks, especially in low-income neighborhoods, is a pressing issue, prioritizing the development of high-quality parks is paramount. Policymakers should consider allocating increased funding for park construction and maintenance, encouraging outdoor physical activity and leisure. Additionally, offering incentives to communities for establishing and maintaining their green spaces, such as community gardens or small parks, through mechanisms like tax breaks or grants, can bolster the availability of outdoor areas and community involvement in promoting healthy lifestyles.

Embedding nutrition and sports education into school curricula is another crucial step. Policymakers can adopt evidence-based programs proven effective in promoting healthy behaviors. These programs might encompass nutrition education integrated into the school curriculum, which has demonstrated positive effects on dietary habits among children and adolescents. Moreover, providing teacher training in delivering nutrition and sports education equips educators to effectively promote healthy lifestyle behaviors among students.

To cultivate healthier eating habits, ensuring healthier food options in school canteens is essential. Policymakers should collaborate with school administrators and food service providers to enact policies limiting the accessibility of unhealthy food choices and championing healthier alternatives. Integrating nutrition education into the school experience equips students with the knowledge to make informed dietary decisions.

Motivating students to engage in physical activity can be achieved through incentives and the creation of a supportive environment. Policymakers can consider implementing rewards for achieving physical activity goals or participating in sports teams, positively reinforcing healthy behaviors. Additionally, fostering safe and accessible outdoor spaces, promoting active transportation to school, and providing extracurricular activities that encourage physical activity contribute to a supportive environment for students.

Promoting physical activity outside of school by encouraging parents to visit parks with their children is vital. Policymakers can forge partnerships with community organizations, such as parent–teacher associations and local recreation centers, to advocate for park use and outdoor play. Offering transportation options to and from parks, especially in areas with limited access, facilitates park visits. Furthermore, investing in park amenities and offering diverse activities like exercise classes and nature walks can enhance the appeal of parks for families. Engaging with the community through surveys and meetings to discern their needs and preferences regarding parks is essential, aligning efforts with the desires of the families policymakers aim to serve.

Ultimately, we anticipate that our study will empower Jordanian policymakers to take effective action in improving nutrition and physical activity, thereby fostering healthier lifestyles among children and adolescents.

## Figures and Tables

**Table 1:** Sociodemographic characteristics of the study participants (*n* = 1040).

Variable	Number (percentage)
Gender	
Male	596 (57.3%)
Female	444 (42.7%)
Nationality	
Jordanian	906 (87.1%)
Other	134 (12.9%)
Residency area	
Amman	890 (85.6%)
Madaba	150 (14.4%)
Chronic illness	
Yes	55 (5.3%)
No	985 (94.7%)
Medication	
Yes	52 (5.0%)
No	988 (95.0%)
Smoking	
Yes	101 (9.7%)
No	939 (90.3%)
Physical activity category	
Low	565 (54.3%)
Moderate	365 (35.1%)
High	110 (10.6%)

**Table 2:** Frequency of perceived barriers to healthy eating among Jordanian adolescents (%), Mean (SD).

Perceived barriers	Very important (%)	Somewhat important (%)	Not important (%)
Personal and environmental barriers to healthy eating	34.0	40.2	25.8
1. I do not have enough information about healthy nutrition	40.2	44.4	15.4
2. I have no motivation to eat healthy food	38.8	41.8	19.3
3. I do not enjoy healthy food	33.8	40.4	25.9
4. There are no nearby places selling healthy food	32.3	41.5	26.2
5. I do not have the skill to plan, buy, prepare, or cook healthy foods	34.3	38.1	27.6
6. I cannot buy healthy food because it is expensive	24.5	35.1	40.4
Social barriers to healthy eating	33.0	35.2	31.9
7. I do not have time to prepare healthy foods because of homework	35	37.3	27.7
8. Parents do not encourage me to eat healthy foods	38.1	28.1	33.8
9. Friends do not encourage me to eat healthy foods	30.6	37.2	32.2
10. Teachers do not encourage me to eat healthy foods	28.1	38.3	33.7
Perceived barriers to healthy eating	33.6	38.2	28.2

**Table 3:** Frequency of perceived barriers to physical activity among Jordanian adolescents (%), Mean (SD).

Barriers	Very important (%)	Somewhat important (%)	Not important (%)
Personal barriers to physical activity	36.2	33.9	29.9
1. I have no motivation for physical activity or sports	38.3	32.6	29.1
2. I do not enjoy physical activity or sports	34.2	33.8	31.9
3. I do not have enough skill to exercise correctly	36	35.4	28.7
Social barriers to physical activity	30.6	37.0	32.4
4. I do not find motivation from parents to exercise	33.8	34.2	31.9
5. I do not find motivation from friends to exercise	31.4	38.3	30.3
6. I do not find motivation from teachers to exercise	26.5	38.4	35.1
Environmental barriers to physical activity	33.6	38.2	28.2
7. I do not find the time to exercise because of homework	35.5	39.4	25.1
8. I do not have enough information on how to increase my physical activity	34.1	37.9	28
9. There is no suitable place for exercise	31.1	37.3	31.6
10. There are no places with suitable prices where I can practice sports	31.3	34.8	33.8
11. The weather is not suitable for exercise	26.2	39	34.8
12. I cannot exercise outside the house due to customs and traditions	25.4	31.4	43.2
13. I feel ashamed when playing sports in front of others	25.2	30.8	44
14. I do not have the financial means to participate in health clubs	23.9	30.7	45.4
Perceived barriers to physical activity	30.9	35.3	33.8

**Table 4:** Frequency of perceived barriers to healthy eating and physical activity in female versus male Jordanian adolescents.

Perceived barriers		Females (*n* = 444)			Males (*n* = 596)			*p* value
		Very important (%)	Somewhat important (%)	Not important (%)	Very important (%)	Somewhat important (%)	Not important (%)	
Personal and environmental barriers to healthy eating	I do not have enough information about healthy nutrition	40.8	46.8	12.4	39.8	42.6	17.6	0.060
	I have no motivation to eat healthy food	39.0	43.0	18.0	38.8	40.9	20.3	0.621
	I do not enjoy healthy food	35.6	39.4	25.0	32.4	41.1	26.5	0.555
	I do not have the skill to plan, buy, prepare, or cook healthy foods	30.0	43.0	27.0	34.1	40.4	25.5	0.375
	There are no nearby places selling healthy food	36.3	39.4	24.3	32.9	37.1	30.0	0.122
	I cannot buy healthy food because it is expensive	24.3	36.0	39.6	24.7	34.4	40.9	0.855

Social barriers to healthy eating	Parents do not encourage me to eat healthy foods	38.3	27.7	34.0	37.9	28.4	33.7	0.973
	Friends do not encourage me to eat healthy foods	29.1	38.3	32.7	31.7	36.4	31.9	0.645
	Teachers do not encourage me to eat healthy foods	26.1	38.1	35.8	29.5	38.4	32.0	0.345
	I do not have time to prepare healthy foods because of homework	36.5	33.6	30.0	33.9	40.1	26.0	0.089

Personal barriers to physical activity	I have no motivation for physical activity or sports	34.9	34.9	30.2	40.8	30.9	28.4	0.146
	I do not have enough skill to exercise correctly	35.8	35.8	28.4	36.1	35.1	28.9	0.968
	I do not enjoy physical activity or sports	34.7	35.1	30.2	33.9	32.9	33.2	0.559

Social barriers to physical activity	I do not find motivation from parents to exercise	35.6	35.4	29.1	32.6	33.4	34.1	0.225
	I do not find motivation from friends to exercise	30.9	38.3	30.9	31.9	38.3	29.9	0.920
	I do not find motivation from teachers to exercise	26.6	39.6	33.8	26.5	37.4	36.1	0.702

Environmental barriers to physical activity	I do not have enough information on how to increase my physical activity	33.1	41.7	25.2	34.9	35.1	30.0	0.072
	There is no suitable place for exercise	32.4	39.6	27.9	30.0	35.6	34.4	0.084
	There are no places with suitable prices where I can practice sports	34.0	34.0	32.0	29.4	35.4	35.2	0.261
	I do not find the time to exercise because of homework	39.6	38.5	21.8	32.4	40.1	27.5	0.027⁣^∗^
	I feel ashamed when playing sports in front of others	25.5	30.4	44.1	25.0	31.0	44.0	0.972
	The weather is not suitable for exercise	24.3	41.0	34.7	27.5	37.6	34.9	0.417
	I cannot exercise outside the house due to customs and traditions	27.7	31.3	41.0	23.7	31.5	44.8	0.287
	I do not have the financial means to participate in health clubs	21.4	31.5	47.1	25.8	30.0	44.1	0.250

⁣^*∗*^*p* value < 0.05 indicates statistical significance in mean perceived barrier between male and female based on the chi-square test.

**Table 5:** Frequency of perceived barriers to healthy eating and physical activity by BMI.

Perceived barriers		Individuals with normal weight (*n* = 730)			Individuals classified as overweight (*n* = 170)			Individuals in the obese category (*n* = 140)			*p* value
		Very important (%)	Somewhat important (%)	Not important (%)	Very important (%)	Somewhat important (%)	Not important (%)	Very important (%)	Somewhat important (%)	Not important (%)	
Personal and environmental barriers to healthy eating	I do not have enough information about healthy nutrition	18.1	43.0	38.9	11.2	48.2	40.6	19.3	33.6	47.1	0.180
	I have no motivation to eat healthy food	20.4	40.1	39.5	15.3	45.9	38.8	18.6	35.7	45.7	0.405
	I do not enjoy healthy food	26.0	39.9	34.1	21.2	42.4	36.5	30.7	28.6	40.7	0.348
	I do not have the skill to plan, buy, prepare, or cook healthy foods	28.1	39.6	32.3	21.2	45.9	32.9	22.1	46.4	31.4	0.214
	There are no nearby places selling healthy food	37.8	35.1	27.1	24.7	44.1	31.2	33.6	18.6	47.9	0.037
	I cannot buy healthy food because it is expensive	34.0	30.7	35.3	42.4	18.1	39.4	44.3	17.1	38.6	0.031

Social barriers to healthy eating	Parents do not encourage me to eat healthy foods	38.9	25.8	35.3	28.8	35.3	35.9	36.4	31.4	32.1	0.115
	Friends do not encourage me to eat healthy foods	33.7	34.7	31.6	28.2	41.8	30.0	32.9	35.7	31.4	0.049
	Teachers do not encourage me to eat healthy foods	33.4	37.2	29.4	31.2	45.9	22.9	31.4	43.6	25.0	0.068
	I do not have time to prepare healthy foods because of homework	25.3	36.0	38.7	25.9	38.8	35.3	37.9	31.4	30.7	0.425

Personal barriers to physical activity	I have no motivation for physical activity or sports	28.8	30.9	40.3	27.6	34.7	37.6	32.9	30.0	37.1	0.115
	I do not have enough skill to exercise correctly	31.8	30.8	37.4	27.6	37.1	35.3	31.4	46.4	22.1	0.574
	I do not enjoy physical activity or sports	31.5	30.2	38.2	35.3	35.9	28.8	37.9	28.6	33.6	0.003

Social barriers to physical activity	I do not find motivation from parents to exercise	30.1	33.6	36.3	32.9	35.3	31.8	38.6	34.3	27.1	0.288
	I do not find motivation from friends to exercise	29.8	40.2	30.0	33.9	32.9	33.2	28.6	38.6	32.9	0.294
	I do not find motivation from teachers to exercise	25.5	36.0	38.5	30.6	31.8	37.6	35.7	25.7	38.6	0.344

Environmental barriers to physical activity	I do not have enough information on how to increase my physical activity	28.1	37.9	34.0	33.5	37.1	29.4	47.1	33.6	19.3	0.061
	There is no suitable place for exercise	31.3	34.1	34.5	34.7	30.6	34.7	44.3	35.0	20.7	≤ 0.001
	There are no places with suitable prices where I can practice sports	23.2	35.6	41.2	25.9	40.0	34.1	30.7	38.6	30.7	0.013
	I do not find the time to exercise because of homework	45.3	28.8	25.8	38.2	33.5	28.2	17.9	38.6	43.6	0.528
	I feel ashamed when playing sports in front of others	45.3	28.8	25.9	35.3	33.5	31.2	43.6	21.4	35.0	0.036
	The weather is not suitable for exercise	34.5	37.4	28.2	35.3	40.0	24.7	35.0	27.9	37.1	0.182
	I cannot exercise outside the house due to customs and traditions	44.8	27.9	27.3	43.6	31.2	25.3	42.1	33.6	24.3	0.973
	I do not have the financial means to participate in health clubs	40.9	30.7	28.4	40.0	33.5	26.5	37.1	30.7	32.1	0.351

*Note:p* value < 0.05 indicates statistical significance in mean perceived barrier between BMI categories based on the chi-square test.

## Data Availability

Data are available upon request.
